# Effect of low oxygen tension on transcriptional factor OCT4 and SOX2 expression in New Zealand rabbit bone marrow-derived mesenchymal stem cells

**DOI:** 10.14202/vetworld.2020.2469-2476

**Published:** 2020-11-18

**Authors:** Erma Safitri

**Affiliations:** 1 Department of Veterinary Reproduction, Faculty of Veterinary Medicine, Universitas Airlangga, Surabaya 60115, Indonesia; 2 Stem Cells Research Division, Institute Tropical Disease, Universitas Airlangga, Surabaya 60115, Indonesia

**Keywords:** bone marrow, cluster differentiation 44, cluster differentiation 90, culture *in vitro*, low O_2_ tension, mesenchymal stem cells, octamer-binding transcription factor 4, pluripotency, sex-determining region Y-box 2

## Abstract

**Background and Aim:**

Octamer-binding transcription factor 4 (OCT4) and sex-determining region Y-box 2 (SOX2) are transcription factors whose functions are essential to maintain the pluripotency of embryonic stem cells. The purpose of this study was to derive stem cells for *in vitro* culture and to maintain their viability and pluripotency, with the goal to obtain a cell line for transplantation in patients with degenerative diseases or injuries. This research focused on examining the effect of low oxygen tension on the ability of bone marrow-derived mesenchymal stem cells (BM-MSCs) to express OCT4 and SOX2 *in vitro*.

**Materials and Methods:**

BM-MSCs were obtained from femurs of 2000 to 3000 g New Zealand male rabbits. BM-MSCs were divided into three groups to test different culture conditions: A control group under hyperoxia condition (21% O_2_) and two treatment groups with low oxygen tension (1% and 3% O_2_). We characterized the BM-MSCs using flow cytometric measurement of cluster differentiation 44 (CD44) and cluster differentiation 90 (CD90) expression. The expression of OCT4 and SOX2 was measured by immunofluorescence staining after 48 h of incubation in chambers with normal or low oxygen tension with controlled internal atmosphere consisting of 95% N_2_, 5% CO_2_, and 1% O_2_ (T1) and 3% O_2_ (T2). We considered OCT4 and SOX2 as two markers of pluripotency induction. All immunofluorescence data were subjected to a post hoc normality Tukey’s honestly significant difference test; all differences with p<5% were considered significant.

**Results:**

BM-MSCs were positive for CD44 and CD90 expression after isolation. Oxygen tension culture conditions of 1% and 3% O_2_ led to OCT4 and SOX2 expression on culture days 2 and 4 (p<0.05), respectively, as compared to the hyperoxia condition (21% O_2_).

**Conclusion:**

Based on the OCT4 and SOX2 immunofluorescence data, we conclude that the stem cells were pluripotent at low O_2_ tension (at 1% O_2_ on day 2 and at 3% O_2_ on day 4), whereas under 21% O_2_ the OCT4 and SOX2 were not expressed.

## Introduction

Pluripotency is the ability of stem cells to differentiate into all cell types that constitute the adult body. Multipotent of rabbit’s bone marrow-derived mesenchymal stem cells (BM-MSCs) can be induced to become pluripotent *in vitro* when cultured under low O_2_ tension conditions [1]. Low O_2_ tension is required to induce a favorable microenvironment to maintain MSCs *in vitro*, mimicking their BM niche [2], to ensure their viability for transplantation [3]. The low O_2_ tension in the BM helps to maintain stem cells adjusted to the body’s normal physiological condition [4]. This maintenance would likely occur if the stem cells are in the G0 phase and not in a cycling state (G1/S/G2/M) [5]. However, MSCs non-proliferative and undifferentiated *in vivo* if there is no injury [6].

MSCs require physiological O_2_ tension levels to be 1% in the BM [7], 10-15% in the adipose tissue, and 2-9% in almost all other tissues [8]. Therefore, to obtain pluripotent characteristics *in vitro*, a low O_2_ tension has to be guaranteed during cell culture procedures to achieve their suitable physiological microenvironment. However, the optimal concentration and duration of low O_2_ tension to induce or maintain pluripotency have not yet been defined. Low O_2_ tension has been used in *in vitro* microenvironments to support several stem cell types: 0-5% O_2_ for hematopoietic stem cells [5]; 2% for adipose stem cells [9], 1-5% for neural stem cells (NSCs) [10], 3% during 7 days for human cord blood cells [11], and 2% for MSCs [12]. Recent studies have focused on searching for the *in vitro* factor combination necessary to control the proliferation of stem cells that would enable them to remain viable and undifferentiated [13] and to prevent apoptosis, senescence, or gene mutations.

In light of the potential therapeutic benefits that can derive from stem cell transplantation in patients with a degenerative disease or injury, understanding how to keep cell viability and pluripotency characteristics *in vitro* are of primordial importance [14]. Common *in vitro* culture conditions rely on hyperoxia (high O_2_ levels), which represents non-physiological environment for stem cells that are used to hypoxia (low O_2_ levels). Moreover, the pluripotency of stem cells *in vitr*o is also affected by the cultivation time under low O_2_ conditions [15]. For example, after 48 h of low O_2_ tension, the role of the transcription factor hypoxia-inducible factor (HIF)-1α is replaced by HIF-2α, which has different target genes [15]. The expression of HIF target genes *in vitro* was suggested to influence the expression of the pluripotency genes [16] octamer-binding transcription factor 4 (OCT4), sex-determining region Y-box 2 (SOX2), NANOG [17], reduced expression (REX-1) [18], Kruppel-like factor 4, and c-MYC [19].

The exact procedure to induce pluripotency in MSCs using low O_2_ tension *in vitro* before stem cell transplantation still remains unexplored. Therefore, this study aimed to determine the O_2_ concentration (comparing 1%, 3%, and 21%) and the culture time (1, 2, 4, and 8 days) required to achieve pluripotency of MSCs *in vitro*.

## Materials and Methods

### Ethical approval

All experimental protocols were reviewed and approved by the Animal Care and Use Committee, the Institutional Animal Ethics Committee (Veterinary Ethical Clearance), Faculty of Veterinary Medicine, Universitas Airlangga.

### Study period and location

All experiments were conducted at Stem Cells Research Laboratory, Institute Tropical Disease, Universitas Airlangga, Surabaya 60115, Indonesia from January to April 2020.

### Animal source of MSCs

Seven-month-old New Zealand male rabbits (±3000 g) were housed in polycarbonate cages with 12-h light-dark cycle at a constant temperature of 21°C. Rabbits were provided from Laboratory animal trial, Institute Tropical Disease, Universitas Airlangga, Surabaya 60115. Adult rabbits were provided a food pellet diet and fresh hay, vegetables, and water *ad libitum*. For the isolation of BM-MSCs, we used one femur from one male rabbit. The sampling technique of the BM-MSCs from the rabbit’s femur was based on Lemeshow’s method [20]. Animals were anesthetized before MSCs isolation.

### Isolation and culture of BM-MSCs

The femur from a male rabbit (±3000 g) was excised and its BM cells were slowly flushed out from the bone cavity. The BM aspirate was transferred to a 15 mL Heparin tube (Thermo Fisher^®^, USA) previously filled with3 mL α-minimum essential medium (Sigma-Aldrich^®^, USA) supplemented with 10% inactivated fetal bovine serum (FBS), 2 mL L-glutamine (Sigma-Aldrich^®^, USA), and 100 U/mL penicillin and streptomycin (Thermo Fisher, USA). The final concentration was 10 mL. The aspirate was then transferred to a 15 mL sterile tube and diluted with 1× sterile phosphate-buffered saline (PBS) (Sigma-Aldrich, USA) to a total volume of 8 mL maintaining a neutral pH. The tube was then rinsed twice with 5 mL PBS and then the aspirate was transferred to Ficoll solution (Sigma-Aldrich^®^, USA) in a separated 15 mL tube. The aspirate was then coated with Ficoll before being centrifuged (Sorvall™ MX Micro-Ultracentrifuge, Thermo Fisher, USA) at 1600 rpm for 15 min at room temperature. After centrifugation, the collection was done from the “buffy coat” location on the surface of the Ficoll-PBS solution using a sterile Pasteur pipette and placed in a 15 mL tube (Thermo Fisher^®^, USA). Each sample was diluted with 1× PBS to a total volume of 15 mL and the tube was turned 3-5 times to achieve an even mix. Next, the sample was centrifuged at 1600 rpm for 10 min, the supernatant was discarded and cells were suspended in 8 mL of cell culture medium before heating. After seeding between 5 cm^2^ and 10 cm^2^ (appropriate 200 million) of cells on the plate, cells were incubated at 37°C and 5% CO_2_ and allowed to attach. Approximately 24 h later, the media and cells that were not attached were discarded and the dish was washed twice with 5 mL pre-heated 1× PBS before 10 mL of fresh cell culture medium was added to the dish for further incubation. The cells were incubated at 37°C and 5% CO_2_ and observed daily using an inverted microscope. Culture media were changed every 3 days. This process was continued until the cells reached a confluency of 80-90%. Forty-eight hours before cell collection, cells were balanced in the incubator at 37°C in a humidified tissue culture incubator containing 5% CO_2_ and 95% air. Non-adherent cells were removed after 24 h and the presence of MSCs was confirmed under an inverted microscope [20].

### Identification of surface markers of BM-MSCs by flow cytometry

BM-MSCs were harvested by centrifugation at the end of the cell culture period and a single-cell suspension was prepared using trypsinization before flow cytometry analysis. Approximately 2×10^5^ cells per sample were washed twice with PBS. Cells were then incubated in test tubes or microtiter plates with unlabeled or fluorescently conjugated antibodies and analyzed by flow cytometry (Becton Dickson FACSVerse, San Diego, USA). The antibodies for surface markers were a PerCP-Cy5.5 anti-human cross anti-rabbit CD45 (Cat # 45-0459-42, Bioscience, Thermo Fisher, USA) and an anti-cluster differentiation 90 (CD90) FITC anti-rabbit CD90 (Bs-0778R, Biossusa, San Diego, USA).

### Low O_2_ tension in MSCs culture

At the third cell culture passage, BM-MSCs were collected and placed on culture dishes with a diameter of 5 cm at a density of 2×10^7^ cells/cm^2^. Iscove’s Modified Dulbecco’s medium was supplemented with 15% FBS (as BM-MSCs stimulator supplement), and antibiotics (100 IU penicillin and 100 μg/mL streptomycin) and was used to culture cells at a temperature of 37°C and with a mixture of 5% CO_2_ and 95% air [21]. Low O_2_ tensions (1 and 3%) [22,23] were achieved by integrating the culture flask into a specialized incubator for low O_2_ tension (Modular Incubator Chamber).

The BM-MSCs at passage three post-isolation were divided into three treatment groups [22,23], each with ten replicates: Control group with BM-MSCs under hyperoxic conditions (O_2_=21%); treatment Group 1 with BM-MSCs under 1% O_2_ tension; and treatment Group 2 with BM-MSCs under 3% O_2_ tension. All groups were cultured for 1, 2, 4, or 8 days.

### Analysis of pluripotency induction

The analysis of the pluripotency induction was based on the identification of OCT4 and SOX2 proteins by immunofluorescence staining [24,25]. For this, MSCs were harvested, collected in a 15 mL tube, and fixed with methanol. After 15 min, rabbit FITC-conjugated anti-OCT4/POU5F1 polyclonal antibody Cat # 45-0459-42, Bioscience, Thermo Fisher, USA) and rabbit FITC-conjugated anti-SOX2 polyclonal antibody (Cat. No bs-0523R-FITC; Biossusa) were added. The samples were washed with PBS, placed on glass slide and then incubated in incubator at 37°C for 1 h so that it adheres and finally analyzed under a fluorescence microscope [21,25].

### Statistical analysis

The immunofluorescence data were analyzed using the IBM SPSS Statistics 20.0 (IBM Corp., NY, USA) and subjected to a *post hoc* normality Tukey’s honestly significant difference test; all differences with p<5% were considered significant.

## Results

**Figure-1:**
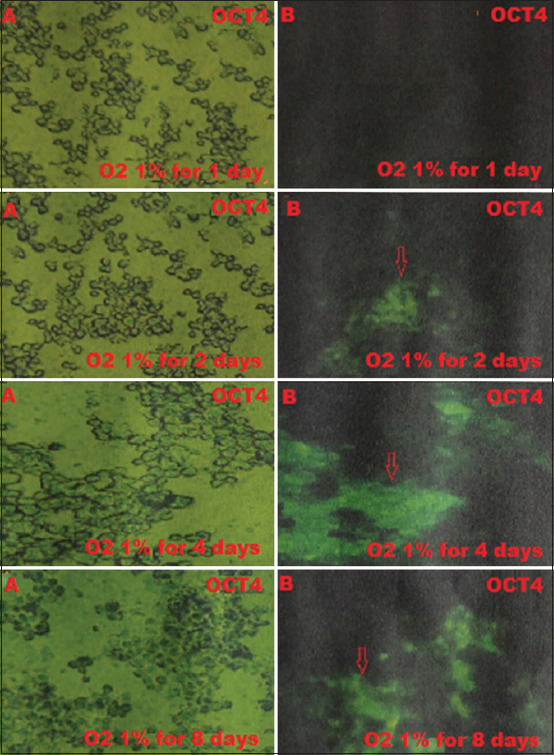
The positive expression on day 2 of the octamer-binding transcription factor 4 (OCT4) is 1% O_2_ in hypoxic precondition treatment group during which cultivation time is 1, 2, 4, and 8 days using fluorescence microscope; (A) without a filter and microscope (400×); (B) containing a green filter, OCT4 positively shows green fluorescence (red arrow).

**Figure-2:**
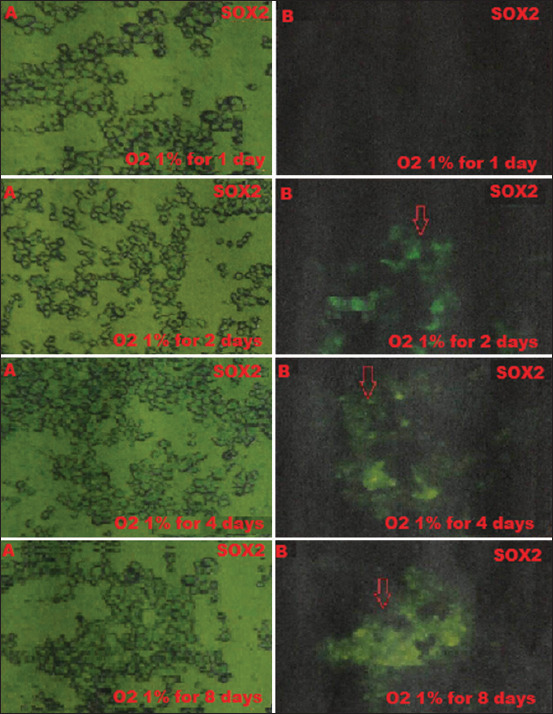
The positive expression on day 2 of sex-determining region Y-box 2 (SOX2) is 1% O_2_ in hypoxic precondition treatment group during which cultivation time is 1, 2, 4, and 8 days using the fluorescence microscope; (A) without filter and microscope (400×); (B) with a green filter, SOX2 positively shows green fluorescence (red arrow).

**Figure-3:**
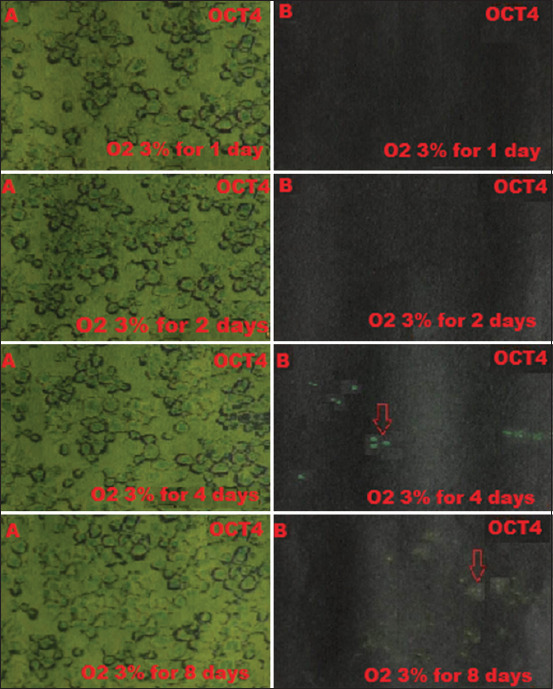
Positive expression on day 4 of the octamer-binding transcription factor 4 (OCT4) is 3% O_2_ in hypoxic precondition treatment group during which cultivation time is 1, 2, 4, and 8 days using the fluorescence microscope; (A) without filter and microscope (400×); (B) with a green filter, OCT4 positively shows green fluorescence (red arrow).

**Figure-4:**
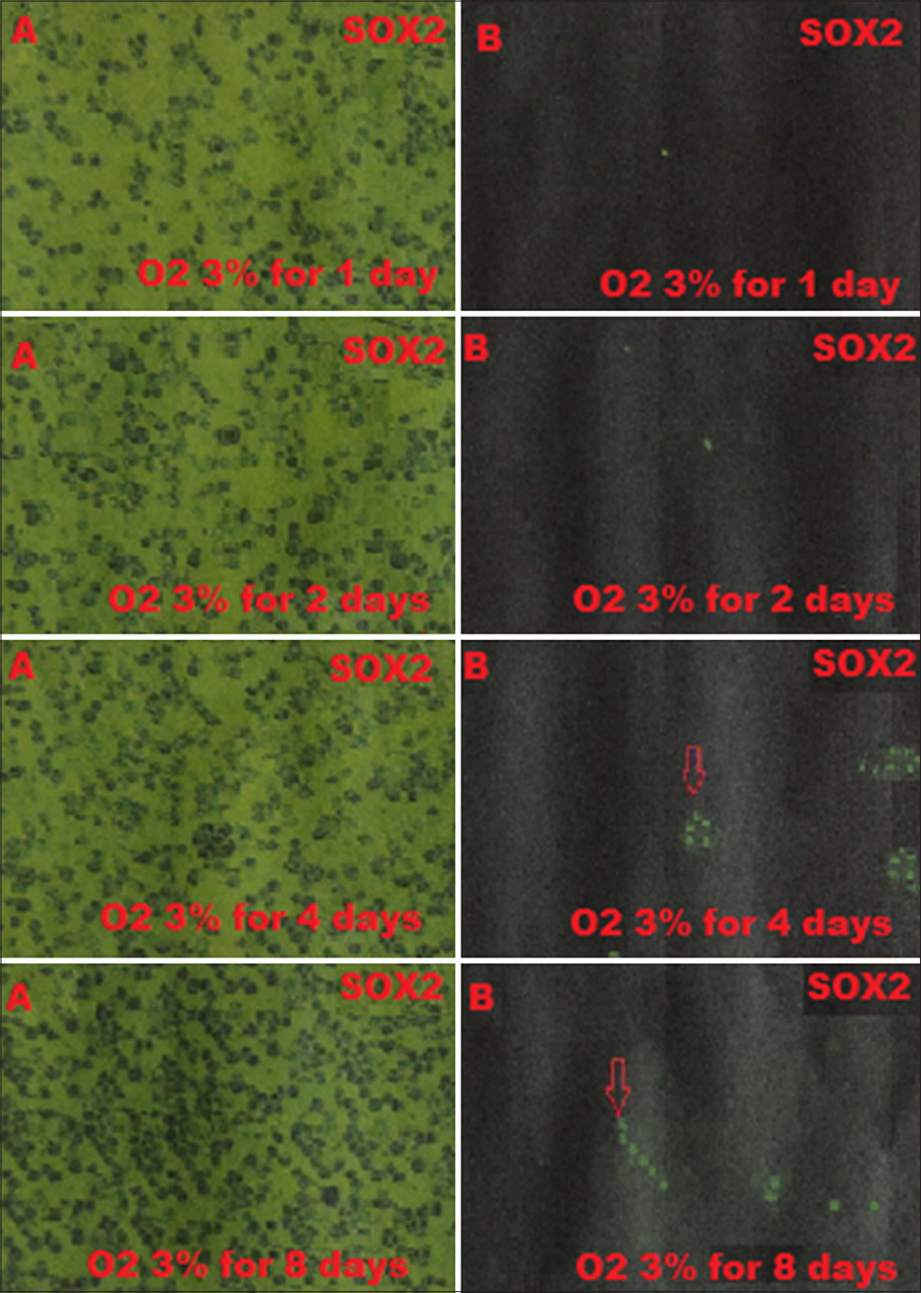
Positive expression on day 4^th^ of the sex-determining region Y-box 2 (SOX2) is 3% O_2_ in hypoxic precondition treatment group during which cultivation time is 1, 2, 4, and 8 days using fluorescence microscope; (A) without filter and microscope (400×); (B) with a green filter, SOX2 positively shows green fluorescence (red arrow).

**Figure-5:**
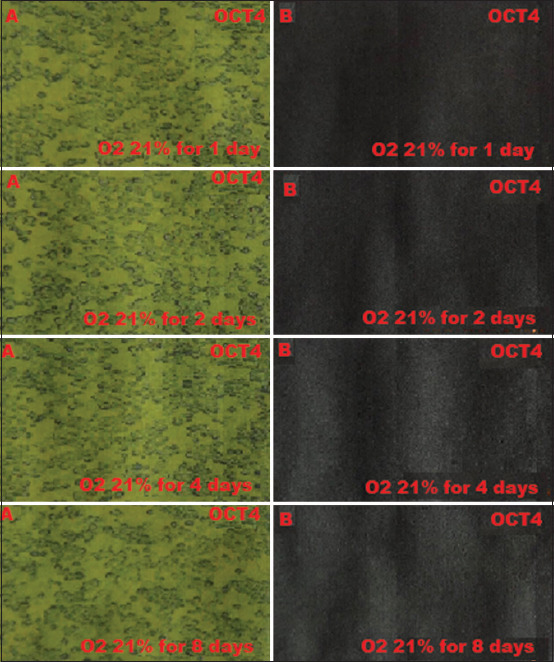
The negative expression of octamer-binding transcription factor 4 (OCT4) in 21% O_2_ normoxia during which cultivation time is 1, 2, 4, and 8 days using fluorescence microscope; (A) without filter and microscope (400×); (B) with green filter, OCT4 negatively does not show green fluorescence.

**Figure-6:**
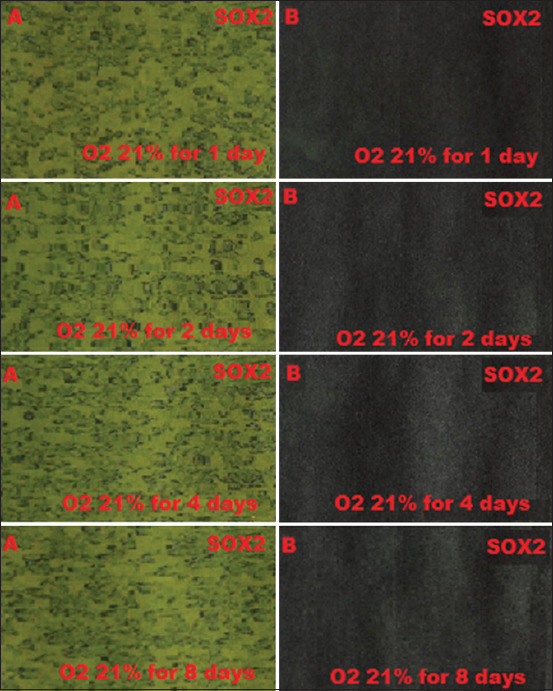
Negative expression of sex-determining region Y-box 2 (SOX2) in 21% O_2_ normoxia during which cultivation time is 1, 2, 4, and 8 days using fluorescence microscope; (A) without filter and microscope (400×); (B) with green filter, SOX24 negatively does not show green fluorescence.

**Figure-7:**
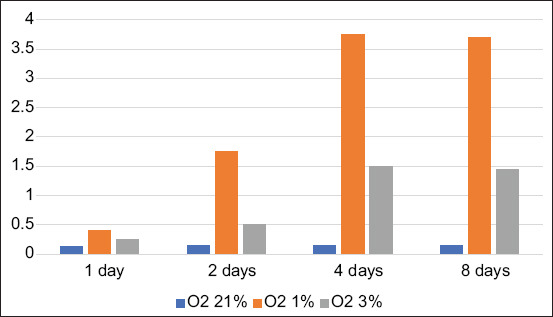
Score of octamer-binding transcription factor 4 in 1, 2, 4, and 8 days in some treatments.

**Figure-8:**
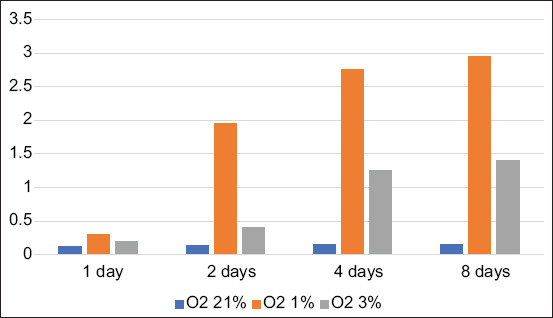
Score of sex-determining region Y-box 2 in 1, 2, 4, and 8 days in some treatments.

BM-MSCs are multipotent stem cells and thus do not express pluripotent cell-specific transcription factors such as OCT4 and SOX2. We tested whether low O_2_ tension could induce rabbit BM-MSCs to become pluripotent *in vitro*. After being cultured under hypoxic (1% or 3% O_2_) or normoxic (21% O_2_) conditions, we characterized the OCT4 and SOX2 expression in BM-MSCs by immunofluorescence. We observed that OCT4 and SOX2 positive cells appeared after 2 and 4 days under 1% and 3% O_2_ tension conditions (Figures-1-4), respectively, whereas under normal O_2_ tension OCT4 and SOX2 expression was not identified (p<0.05) (Figures-5 and 6). Of note, the fluorescence intensity on day 4 at 3% _O_2 tension was lower than on day 2 at 1% _O_2 tension (p<0.05) (Tables-1 and 2 or Figures-7 and 8 for complete immunofluorescence quantification). This suggests that the hypoxic conditioning led to the expression of transcription factors potentially enabling the BM-MSCs to become pluripotent. Furthermore, this observation indicates that the cultivation time under a specific O_2_ tension also plays an important role in the timing of OCT4 and SOX2 expression after isolation.

**Table-1:** Score of OCT4 in 1, 2, 4, and 8 days in some treatments.

Treatments	Average OCT4 expression score±SD in 1 day	Average OCT4 expression score±SD in 2 days	Average OCT4 expression score±SD in 4 days	Average OCT4 expression score±SD in 8 days
Conventional/ Hyperoxia O_2_ 21% (C)	0.13^a^±0.50	0.14^a^±0.35	0.15^a^±0.60	0.15^a^±0.50
Low O_2_ tension O_2_ 1% (T1)	0.40^a^±0.35	1.75^b^±0.55	3.75^c^±0.45	3.70^c^±0.40
Low O_2_ tension O_2_ 3% (T2)	0.25^a^±0.50	0.50^a^±0.35	1.50^b^±0.30	1.45^b^±0.50

^a,b,c,d^Different superscripts in the same column or row were significantly different (p<0.005). OCT4=Octamer-binding transcription factor 4, SD=Standard deviation

**Table-2:** Score of SOX2 in 1, 2, 4, and 8 days in some treatments.

Treatments	Average SOX2 expression score±SD in 1 day	Average SOX2 expression score±SD in 2 days	Average SOX2 expression score±SD in 4 days	Average SOX2 expression score±SD in 8 days
Conventional/Hyperoxia O_2_ 21% (C)	0.12^a^±0.40	0.14^a^±0.50	0.15^a^±0.40	0.15^a^±0.30
Low O_2_ tension O_2_ 1% (T1)	0.30^a^±0.40	1.95^b^±0.45	2.75^c^±0.35	2.95^c^±0.50
Low O_2_ tension O_2_ 3% (T2)	0.20^a^±0.50	0.40^a^±0.40	1.25^b^±0.40	1.40^b^±0.55

^a,b,c,d^Different superscripts in the same column or row were significantly different (p<0.005). SOX2=Sex-determining region Y-box 2, SD=Standard deviation

Overall, this result shows that the treatment with low O_2_ tension leads to the expression of the pluripotency transcription factors OCT4 and SOX2 in otherwise multipotent BM-MSCs, which may indicate that some cells were induced to become pluripotent.

## Discussion

Our analysis of pluripotency based on OCT4 and SOX2 immunofluorescence. The study found that human ESC cultured for 48 h under low O_2_ conditions expressed HIF2-α, which was shown to be an upstream regulator of OCT4 and therefore essential for the maintenance of pluripotency. Interestingly, other transcription factors, such as SOX2 and NANOG, are also regulated by HIF2-α [15]. Furthermore, OCT4, SOX2, and NANOG maintain stem cell characteristics and suppress the expression of differentiation genes.

The relationship between hypoxia, HIF2-α, and pluripotency genes was previously highlighted by a study showing that the reprogramming efficiency during iPS cell generation from mouse embryonic fibroblasts was higher under hypoxia than under normoxia [26,27]. Furthermore, a study describing NSCs transplantation proposed that preconditioning under a hypoxic environment is necessary during cells *in vitro* maintenance [28]. This is in line with the high reliance of NSCs on low O_2_ during early development [29]. The therapeutic efficacy of stem cell transplantation requires adequate knowledge of the mechanisms that affect stem cells during their *in vitro* maintenance, such as migration, proliferation, commitment, and dependence on oxygen levels in the appropriate culturing conditions.

Hypoxic culture conditions were previously linked to the intracellular stabilization pathway of HIF transcription factors. Under low oxygen tension, the α subunit of the HIF (HIF-α) becomes stable and dominant due to the absence of Proline hydroxylase activity [30], which under normoxia acts as a HIF inhibiting factor through oxygen-dependent hydroxylation of a conserved asparagine in the COOH-terminal transactivation domain (CAD) of HIF-α. When oxygen is not available, the asparagine residue is not hydroxylated and transcriptional co-activators such as p300/CBP-CREB (cAMP-response element-binding protein) can interact with CAD, triggering the complete activity of the cell’s response to hypoxia through the stabilization of HIF1-α. After HIF1-α is stabilized, it translocates from the cytoplasm to the nucleus and undergoes a dimerization with HIF1-ß [31], also called ARNT. The dimerization between HIF-1α and HIF1-ß forms the HIF complex and requires the bHLH domain and parts of the periodic acid-Schiff (PAS) domain. The bHLH domain was described to be also important for the binding of the HIF complex to DNA [32] in specific DNA sequences, which are referred to as hypoxia response elements (HREs; 5’-TACGTG-3’), activating the expression of target genes. Of note, the N-terminal transactivating domain confers target gene specificity to HIF [33].

Conversely, under normoxic conditions, cells trigger the degradation pathway of HIF-1α through the oxygen-sensitive hydroxylation of the HIF1-α subunit [34]. HIF1-α contains many proline (Pro) amino acid residues, namely, in the oxygen-dependent degradation domains, which are hydroxylated by propyl hydroxylases (PHDs). PHDs catalyze this reaction through the addition of oxygen in the Pro-402 and Pro-564 residues turning them into 4-hydroxyproline. Oxygen and 2-oxoglutarate are required for the activity of three PHD isoforms (PHD1, PHD2, and PHD3), which have an equally high dependence on oxygen [34]. Following hydroxylation, HIF-1α is destabilized, ubiquitinated, and then degraded by the proteasome [35]. According to Vriend and Reitter [36], the von-Hippel–Lindau E3 ubiquitin ligase, tumor suppression, plays an important role in the regulation of the ubiquitination process of HIF-1α by binding to its hydroxylation subunit. Moreover, this study showed that HIF-1α degradation culminates in the absence of OCT4 and SOX2 expression in culture of BM-MSCc.

Our study infers the pluripotency activity in BM-MSCs after low O_2_ treatment, which is one of the main research focuses on the adult stem cells field.The balance between differentiation, apoptosis, and self-renewal, characteristic of stem cells, needs to be achieved through the regulation by the microenvironmental niche where the stem cells are located. According to Mohyeldin *et al*. [37], in feeder-free *in vitro* culture conditions, the fate of the stem cells is affected by growth factors, interleukins, and serum composition, but is also influenced by the oxygen levels used for cell culture.

Pluripotency, the ability of a cell to differentiate into any cell belonging to the three embryonic layers (ectoderm, mesoderm, and endoderm), is generally an exclusive characteristic of ESCs. Compared to more committed progenitor cells, stem cells, in particular, ESCs possess a higher differentiation potential [13]. ESCs are derived from the inner cell mass of the blastocyst and are easy to differentiate *in vitro* into various types of cells, including nerve, blood, cardiac, and immune cells. In this study, we show that MSCs, adult stem cells that are generally defined as multipotent, can express OCT4 and SOX2 genes after being cultured under low O_2_ tension. In the future, we expect to confirm that MSCs cultured under these *in vitro* conditions have a pluripotent potential and are also fully capable of differentiating into different cell types. This hypothesis is based on other studies, which show high efficiency of cell reprogramming to iPS cell state under low O_2_ tension conditions [26].

## Conclusion

Based on immunofluorescence measurements, we suggest that some BM-MSCs have pluripotency characteristics since they express OCT4 and SOX2 at low O_2_ tension (after 2 days under 1% O_2_ and after 4 days under 3% O_2_ tension), whereas at 21% O_2_ tension OCT4 and SOX2 were not expressed. Thus, we conclude that *in vitro* low oxygen tension conditions increase OCT4 and SOX2 expression (p<0.05) as compared to conventional or hyperoxic conditions.

## Author’s Contributions

ES: Conceptualization, methodology, data analysis, research and ethical clearance preparation of the research equipment, observation of Immunofluorescence method (OCT4 and SOX2), draft for manuscript preparation. Preparation of animal experimental and corresponding author. She has read and approved the final manuscript.
